# Microfluidic-Based 3D Engineered Microvascular Networks and Their Applications in Vascularized Microtumor Models

**DOI:** 10.3390/mi9100493

**Published:** 2018-09-27

**Authors:** Xiaolin Wang, Qiyue Sun, Jianghua Pei

**Affiliations:** 1 Department of Micro/Nano Electronics, School of Electronic Information and Electrical Engineering, Shanghai Jiao Tong University, Shanghai 200240, China; kizluy@sjtu.edu.cn (Q.S.); Peijianghua@sjtu.edu.cn (J.P.); 2 National Key Laboratory of Science and Technology on Micro/Nano Fabrication, Department of Micro/Nano Electronics, School of Electronic Information and Electrical Engineering, Shanghai Jiao Tong University, Shanghai 200240, China; 3 Key Laboratory for Thin Film and Microfabrication Technology (Ministry of Education), Department of Micro/Nano Electronics, School of Electronic Information and Electrical Engineering, Shanghai Jiao Tong University, Shanghai 200240, China

**Keywords:** microfluidics, vascularization, organ-on-a-chip, vascularized tumor model, tissue engineering

## Abstract

The microvasculature plays a critical role in human physiology and is closely associated to various human diseases. By combining advanced microfluidic-based techniques, the engineered 3D microvascular network model provides a precise and reproducible platform to study the microvasculature in vitro, which is an essential and primary component to engineer organ-on-chips and achieve greater biological relevance. In this review, we discuss current strategies to engineer microvessels in vitro, which can be broadly classified into endothelial cell lining-based methods, vasculogenesis and angiogenesis-based methods, and hybrid methods. By closely simulating relevant factors found in vivo such as biomechanical, biochemical, and biological microenvironment, it is possible to create more accurate organ-specific models, including both healthy and pathological vascularized microtissue with their respective vascular barrier properties. We further discuss the integration of tumor cells/spheroids into the engineered microvascular to model the vascularized microtumor tissue, and their potential application in the study of cancer metastasis and anti-cancer drug screening. Finally, we conclude with our commentaries on current progress and future perspective of on-chip vascularization techniques for fundamental and clinical/translational research.

## 1. Introduction

The circulatory system plays a vital role to maintain homeostasis in the human body. It comprises a closed network of arteries, veins, and capillaries that allow blood to circulate throughout the body, not only for waste product removal, but also for gas exchange and nutrient transportation, all of which are essential for organ viability. Besides participating in metabolic function, microvasculature in different organ microenvironment has unique biological functions and physical properties, such as maintaining solute and water balance between the blood and tissue compartments, or responding to different deformations and stress fluctuations [1]. Recently, the concept of “organ-on-a-chip” has been proposed to establish in vitro models that can mimic the microphysiological function and three-dimensional (3D) microstructure of human organ more accurately and specifically compared to the traditional two-dimensional (2D) cultures and animal models [2]. In addition to supplying nutrient and oxygen to the cultured tissue by perfusing the culture medium, vascularization of organ-on-a-chip can also contribute to the establishment of organ-specific microenvironments and microphysiological function by constructing the microvascular with selective barrier function similar to that in vivo. In other words, to better mimic the characteristics and functions of specific human organs in vitro, it is necessary to integrate a perfusable and functional 3D microvasculature to different organ-on-a-chip systems. Microfluidic technologies have emerged as useful tools for the development of organ-on-a-chip, which can offer precise control over various aspects of the cellular microenvironment such as a different profile of fluid flow, gradient of various growth factors, and mechanical properties of versatile biomaterials. All these advantages can facilitate the formation of biomimicking in vitro vascularized microtissue models.

Furthermore, besides the substantial physiological research on constructing and characterizing the microvasculature in homeostatic conditions in these systems, there is a great potential to model pathological conditions to study vascular-related diseases [3]. Especially for cancer biology, the tumor vasculature plays a critical role in several key events in the metastatic cascade, such as intravasation and extravasation. Engineered microvessels can be well suited to the study of mechanisms of tumor growth and metastasis, drug screening, and cancer therapies by establishing the vascularized microtumor models in vitro.

In this review, we focus on the generation of microvascular networks in 3D engineered tissue constructs and their integration into vascularized microtumor models by combining microfluidics, microfabrication, biomaterials, and tissue engineering technologies. We first discuss the current strategies for tissue vascularization. Next, we highlight relevant factors that induce vascularization inside microfluidic systems. We then provide a brief introduction of selective vascular barrier properties in different human organs and methods to construct and characterize these properties. Then, we review the current, state-of-the-art in vitro vascularized tumor-on-a-chip models in various disease stages, and their potential applications for anti-cancer drug screening. Finally, we conclude with our visions to improve the current approaches to create vascularized microtissues. This review will provide a better understanding of the vascularization process for organ-on-a-chip systems and its applications in cancer biology.

## 2. In Vitro Vascularization Strategies

In many early studies to understand the basis of vascular biology, 2D models were constructed by plating endothelial cells (ECs) on a flat surface such as Petri dish [4], porous membrane [5], or patterned hydrogel [6] to form a confluent monolayer to mimic the blood vessel wall. However, these 2D models cannot replicate well the proper physical structure of blood vessel in vivo, and of note is its circular shape and polarized luminal/abluminal surfaces. It is known that the blood vessel in human body is with circular cross-section. In order to mimic the microvascular in vitro, it is necessary to replicate not only its microphysiological function, but also its 3D microstructure (i.e., circular cross-section). In addition, blood vessel polarization in the apical (luminal) to basal (abluminal) axis is important for directed secretion of proteins, which can induce cord hollowing by changing the cell shape to form a lumen [7]. Thus, it is challenging to use these 2D models to replicate luminal flow in vivo and study its effect on vasculature. Due to the advantages of highly regulated spatial and temporal control over cell patterning, chemical gradients, and mechanical stimuli, microfluidics-based techniques have been widely utilized to create 3D models in vitro [8,9]. Besides that, microfluidics also enable the continuous perfusion of cell culture medium to supply the oxygen and nutrient as well as the removal of waste product, which is the necessary condition to realize the long-term survival of these vascularized microtissue. In addition, with the combination of versatile biomaterials and tissue engineering techniques, many 3D in vitro vascularization strategies have been developed, which can be broadly categorized into three main types: (1) EC lining-based methods, (2) vasculogenesis and angiogenesis-based methods, and (3) hybrid methods.

### 2.1. EC Lining-Based Methods

Briefly, the EC lining methods are achieved by allowing EC to form a monolayer on the inner walls of the microfluidic channel. The major advantage of these methods is that the microvasculature geometry and dimensions can be easily controlled based on different microfabrication techniques with high flexibility. Furthermore, shear stress imposed on the microvessels can be precisely controlled and calculated based on the channel dimensions and the applied flow rate. However, these methods cannot mimic vascular formation in vivo that mainly relies on the natural process of vasculogenesis and angiogenesis to form the complex microvascular networks. Since it is difficult to evenly distribute a high density of ECs inside the microfluidic channels with small diameter due to the blockage, EC lining methods are only suitable to construct large blood vessels with diameter greater than 50 μm [10]. The hollow microstructure (i.e., a single channel or a channel network) can be made of either hydrogel or polydimethylsiloxane (PDMS). For hydrogel-based microchannel, various micro-molding methods reported in the literature so far can be grouped into three main types: (1) Microneedle-based removable method for a single microchannel, (2) micropatterned, planar hydrogel slab bonding method for single-layer microchannel network, and (3) dissolvable material-based sacrificial micromolding method for multi-layer microchannel network. For EC lining inside a PDMS-based microfluidic channel, since cells might not adhere tightly to PDMS surface, a thin coating layer of basement membrane proteins (e.g., laminin, fibronectin, collagen IV, etc.) onto the microchannel inner walls is needed to enhance cell adherence.

#### 2.1.1. Microneedle-Based Removable Method

**Figure 1:**
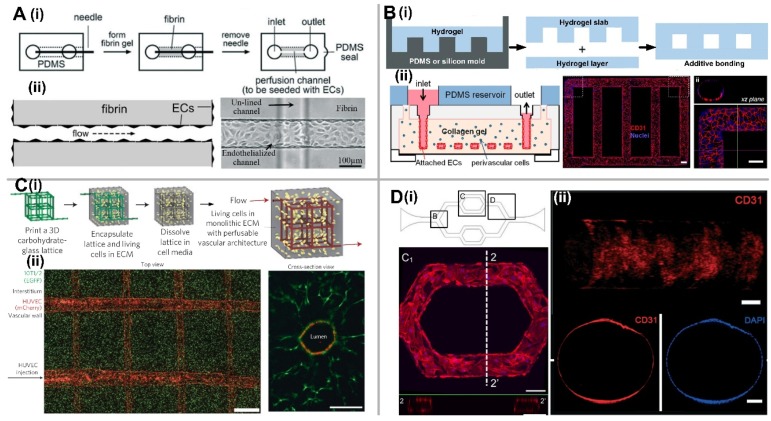
EC lining-based methods for in vitro vascularization. (**A**) Microneedle-based removable method. (**i**) Schematic of the simple needle-molding technique to create fluidic channels inside hydrogels; (**ii**) Endothelialized microchannel under the effect of fluidic flow. Adapted by permission from Reference [13], copyright Wiley Periodicals, Inc. 2012. (**B**) Micropatterned planar hydrogel slab bonding method. (**i**) Schematic of fluidic hydrogels fabricated by micromolding from a PDMS or silicon mold followed by a bonding process; (**ii**) Microvessel network shape inside collagen gel constructed from a micropatterned silicon stamp, and Z-stack projection of horizontal confocal sections of endothelialized microfluidic vessels immunostained with CD 31 from the view of overall network and the corner. Scale bar, 100 μm. Adapted by permission from Reference [17], copyright National Academy of Sciences, USA 2012. (**C**) Dissolvable materials-based sacrificial micromolding method. (**i**) Schematic of a 3D interconnected microvessel network formed by rapid casting of carbohydrate glass lattice as the sacrificial element with a 3D printer. The lattice is encapsulated in ECM along with living cells and dissolved in minutes in cell media without damage to nearby cells, which can generate a monolithic tissue construct with a vascular architecture. (**ii**) The micrograph (left) shows HUVECs expressing mCherry attached to the hydrogel wall to generate the microvessel network. Scale bar, 1mm. The micrograph (right) shows endothelial monolayer lined vascular lumen surrounded by 10T1/2 cells after 9 days in culture. Scale bar, 200 μm. Adapted by permission from Reference [18], copyright Nature Publishing Group 2012. (**D**) EC lining inside a PDMS-based microfluidic channel. (**i**) The schematic of PDMS-based microfluidic channel, and the confocal image of endothelial cell at location C of the channel with rectangle cross section. Scale bar, 100 μm. Adapted by permission from Reference [27], under the Creative Commons Attribution License. (**ii**) Confocal reconstruction image showing the complete lumen formed by the HUVECs inside the PDMS tube, and confocal fluorescence micrographs of the cross-sectional views of an endothelialized PDMS tube stained with CD31/nuclei. Scale bar, 100 μm (top) or 200 μm (bottom). Adapted by permission from Reference [33], copyright Royal Society of Chemistry 2016.

The basic operation procedure of this method is to insert a cylindrical object (e.g., microneedle, wire, capillary tubes, etc.) into a hydrogel solution and remove it after the hydrogel is fully polymerized to create a single microchannel. Then ECs are seeded onto the inner surface of the microchannel to form a confluent EC monolayer (Figure 1A) [11,12,13]. Briefly, this method typically requires two separate steps: Creating a microchannel, and lining it with ECs. Furthermore, Sadr et al. proposed a new method to realize a rapid one-step engineering of microtubular constructs that combined self-assembled monolayer-based cell transfer and hydrogel photocrosslinking techniques [14]. After UV crosslinking, the gold sputtered rod modified with oligopeptide was removed from the polymerized hydrogel by applying electrical stimulation, which would transfer the layer of human umbilical vein endothelial cells (HUVECs) to 3D geometrically defined vascular-like structures in hydrogels. Moreover, a bilayer vascular structure of smooth muscle cells (SMCs) and HUVECs could be formed by a sequential deposition of SMCs, fibronectin as the cell adhesive layer and HUVECs, which exhibited stronger barrier function than EC monolayer [15]. However, since the cylindrical objects need to be removed from hydrogel, this method is limited to a simple, single straight microvascular channel [12,16].

#### 2.1.2. Micropatterned Planar Hydrogel Slab Bonding Method

Rather than a single microchannel, a single-layer microchannel network can also be fabricated by using standard lithographic patterning method. Zheng et al. developed an interconnected microvascular array by using the additive bonding between a micropatterned collagen gel slab and a flat hydrogel layer (Figure 1B). Briefly, a micropatterned collagen gel slab was imprinted from a microstructured PDMS stamp, and bonded to a flat collagen substrate to form a collagen-based microchannel network. Then, ECs were seeded onto the inner wall of these collagen microchannels. Using this model, several vascular biology studies were performed, including vessel sprouting, interaction between ECs and mural cells, endothelial barrier function under different flow conditions, and EC response to various biochemical signals [17]. Compared with the microneedle-based removable method, although 2D planar networks can be easily formed, they usually have the rectangular cross-section due to the inherent characteristics of templates produced by lithographic methods.

#### 2.1.3. Dissolvable Materials-Based Sacrificial Micromolding Method

In this method, a multi-layer microchannel network can be fabricated by dissolving or melting the dissolvable gel or solid material embedded in a gel matrix. With the development of 3D printing technology, more complex multi-layer microchannel networks can be interconnected using a wide range of template materials, such as carbohydrate glass [18], Pluronic F127 [19], agarose [20], gelatin [21], sodium alginate [22], and synthetic polyethylene glycol (PEG) [23]. Miller et al. created a multi-layer microvessel network from the carbohydrate glass template that can provide the sufficient mechanical support in an open lattice (Figure 1C) [18]. After dissolving the carbohydrate glass with cell culture medium, the perfusable, hollow, cylindrical network structure was left inside fibrin gel. HUVECs are then seeded into these hollow microstructures to form a 3D vascular network. While this strategy has the potential to create a complex 3D microvascular network, it is time-intensive and still limited to larger vessels determined by the resolution of 3D printer.

#### 2.1.4. EC Lining inside a PDMS-Based Microfluidic Channel

The PDMS-based microfluidic chips enable a single-layer microchannel network design with the diameter ranging from 60–200 μm, and different microenvironment factors (e.g., flow profile, shear stress, etc.) can be precisely controlled with the sophisticated microfluidic technologies [24]. In vivo, ECs are attached to a basement membrane with the thickness of 40–120 nm [25]. Therefore, for ECs to adhere to a PDMS-based microchannel, extracellular matrix (ECM) proteins like laminin, fibronectin, or collagen IV are required to be deposited onto the microchannel inner wall prior to lining it with ECs (Figure 1(Di)) [26,27]. Moreover, microchannels fabricated through soft lithography technology have the inherent rectangular or square cross section, which cannot closely mimic real microvessels in vivo with a circular cross section. However, Esch et al. showed that ECs could be grown within both square and semicircular microchannels [28]. It was found that shear force has a greater impact on these endothelial structures than geometry, and the channel geometry only influences the final cross-sectional profile of the vascular lining.

To solve the problem of non-circular cross section, Bischel et al. developed a “viscous finger patterning” method that took advantage of basic fluidic principles to create 3D lumens with circular cross section inside a PDMS-based microchannel [29,30]. Briefly, culture medium was pumped into a partially polymerized hydrogel at the central position of the microchannel, creating a circular channel at the center while filling the rectangular corner with hydrogel. Similarly, circular PDMS microchannel can also be fabricated by introducing a pressurized air stream inside a partially solidified rectangular microchannel filled with liquid PDMS or liquid silicone oligomer, followed by baking the device to fully cure the coated layer [31,32]. Furthermore, Zhang et al. developed a hollow PDMS tube with adjustable diameters and wall thickness to mimic the elastomeric free-form blood vessels after EC lining, which could potentially replace inert plastic tubes to integrate multiple organoids into a single microfluidic circuitry (Figure 1(Dii)) [33].

### 2.2. Vasculogenesis and Angiogenesis-Based Methods

**Figure 2:**
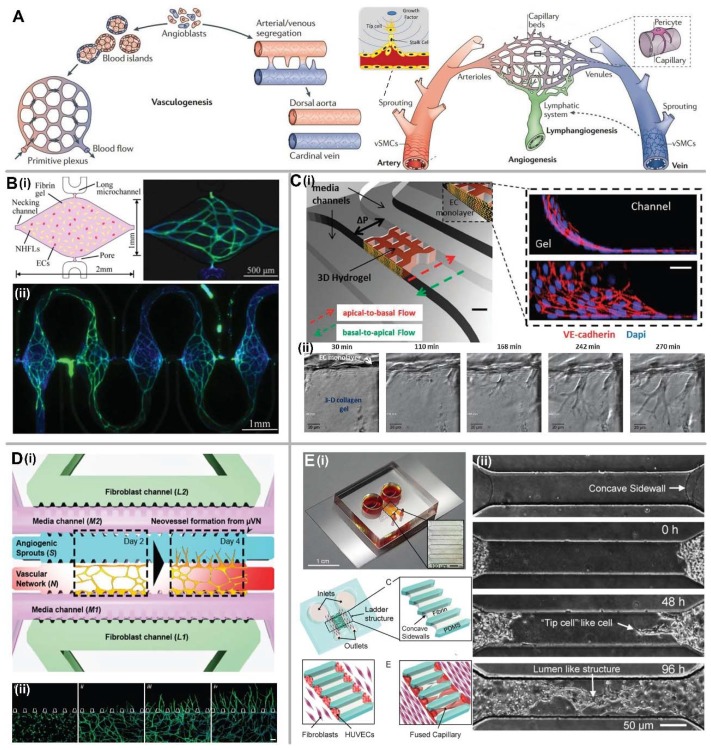
Vasculogenesis and angiogenesis-based methods for in vitro vascularization. (**A**) Schematic of vasculogenesis and angiogenesis in vivo. Adapted by permission from Reference [36], copyright Nature Publishing Group 2011. (**B**) 3D microvascuar network formation by vasculogenesis. (**i**) Schematic of 3D microtissue chamber seeded with ECs and NHLFs in fibrin gel, and formed microvascular network labeled with CD31 (green) and 4′,6-diamidino-2-phenylindole (DAPI) (blue) for nuclei. (**ii**) Fluorescent image of 5 interconnected microvascular networks. Adapted by permission from Reference [39], copyright Royal Society of Chemistry 2013. (**C**) Angiogenesis from EC monolayer. (**i**) Schematic of microfluidic-based 3D cell culture system, and the confocal images of confluent EC monolayer showing coverage on gel and channel surfaces immunostained with anti-VE-cadherin (red) and nuclei (blue). Scale bar, 500 μm. (**ii**) Time-lapse images of sprouting angiogenesis induced by transendothelial flow. Scale bar, 20 μm. Adapted by permission from Reference [48], copyright Royal Society of Chemistry 2012. (**D**) Angiogenesis from 3D microvascular network. (**i**) Configuration of the microfluidic device and schematic of the angiogenesis assay from microvascular network. (**ii**) Temporal sequence of interconnected vascular network formation and angiogenic sprouting from the formed network inside the adjacent microchannel. Scale bar, 100 μm. Adapted by permission from Reference [51], copyright Royal Society of Chemistry 2016. (**E**) Anastomosis of sprouts into interconnected and perfusable blood vessels. (**i**) Schematic of microfluidic device design and self-organized capillary networks formed by HUVECs attached on the concave sidewalls from both sides. (**ii**) Images of sprouting HUVECs from both sides and fused capillary formation over time. Adapted by permission from Reference [52], copyright Royal Society of Chemistry 2012.

Vasculogenesis and angiogenesis are the fundamental processes of vascular development in vivo [34,35], as shown in Figure 2A. Vasculogenesis gives rise to formation of the first primitive vascular plexus during early embryogenesis, while angiogenesis is responsible for the remodeling and expansion of the vascular network [36]. In adults, formation of new blood vessels may occur via vasculogenesis through recruitment of ECs from bone marrow [37], as well as through angiogenesis stimulated by distress signals from the parenchymal tissue under certain physiological and pathological conditions such as wound healing, exercise, and tumor growth [38]. Different from EC lining-based methods, vasculogenesis, and angiogenesis-based methods do not require additional microstructures to guide ECs into forming vascular structure. It allows the seeded cells inside ECM to self-assemble into a 3D microvascular network *de novo*. Therefore, these methods can create a more natural vascular structure than the EC lining methods.

#### 2.2.1. Vasculogenesis

Vasculogenesis is a process in which new blood vessels are formed from endothelial progenitor cells (EPCs) *de novo*. Within a microfluidic device, Hsu et al. demonstrated formation of microvascular networks using endothelial colony forming cell-derived endothelial cells (ECFC-ECs) and normal human lung fibroblast (NHLF) mixed in fibrin gel, and embedded inside a series of diamond-shaped tissue chambers (Figure 2B). Under a physiological level of interstitial flow, a continuous and perfusable microvessel network formed after three weeks by perfusing the cell culture medium in absence of vascular endothelial growth factor (VEGF) and basic fibroblast growth factor (bFGF) [39,40,41]. It was reported that the vascularized microtissue could be cultured and remained viable for up to 40 days and showed disruption and shedding over time [41]. Furthermore, it was found that combination of fibroblast-derived proteins would promote EC sprouting and was necessary for EC lumen formation [42]. VEGF and bFGF can also be utilized as chemical factors to stimulate the vasculogenesis. Raghavan et al. presented a method to control the microvessel geometry by spatially patterning ECs within micromolded collagen gels, and demonstrated that the lumenized microvessels formed within 24–48 h by stimulating with VEGF and bFGF [43]. With this method, various microvessel size and shape can be achieved by modifying the collagen gel concentration and the seeded cell density. It was found that both the average branch length and effective diameter decreased with the increase in fibrinogen concentrations, while the branch length, diameter, and area fraction of the vascularized region all increased with the increase of EC seeding density [44]. Beyond these, paracrine signaling by co-culturing with other types of cells can also influence vascular morphology, such as the number of branches, average branch length, percent vascularized area, and average vessel diameter. It was found that the larger coverage area and network stability can be achieved by co-culturing with fibroblasts, while the diameter and average vessel length are reduced under the effect of angiogenic growth factors [45]. Furthermore, besides the co-culture with fibroblast, the other stromal cells like pericytes, SMCs, or mesenchymal stem cells (MSCs) can contribute the vascular stabilization [46]. These findings will provide mechanisms to fine-tune microvascular network in vitro with specified morphological properties.

#### 2.2.2. Angiogenesis

Angiogenesis is the formation of new blood vessel from pre-existing vessels, which is an important mechanism for vascular network remodeling. Angiogenesis occurs through a series of defined steps, including ECM degradation by matrix metalloproteinases (MMPs), angiogenic stimulus, sprouting, elongation and branching, lumen formation, anastomosis, and finally stabilization or regression [47]. Within microfluidic device, angiogenesis can be induced from either EC-lined microvessel or vasculogenesis-derived microvascular network. After applying the transendothelial flow, the sprouting angiogenesis could be induced from an EC monolayer. It was found that focal adhesion kinase-mediated signaling accompanied by extensive remodeling of cell–cell junctions and redistribution of the actin cytoskeleton contributed to the effect of transendothelial flow on vascular sprouting (Figure 2C) [48]. Furthermore, angiogenic sprouting from the EC monolayer can also be induced by VEGF gradient [49,50]. In addition, Kim et al. developed a 3D microfluidic in vitro model to investigate angiogenic sprouting from vasculogenesis-derived microvascular networks in the presence of interstitial flow (Figure 2D) [51]. It was found that angiogenic sprouting was only promoted in the direction opposite to that of interstitial flow and suppressed in the direction of flow.

To form a perfusable microvascular network, these sprouts need to connect with each other through a process called anastomosis. Yeon et al. established an array of perfusable microvessels from EC monolayer lined on both sidewalls of fibrin gel confined inside a microfluidic channel (Figure 2E) [52]. By co-culturing with lung fibroblasts, the angiogenic sprouts were stimulated and fused together after 3–4 days. The spacing of the angiogenic sprouts can be well controlled by adjusting the spacing of microstructures that confine the ECM during the microfabrication process. Similarly, Song et al. demonstrated a microfluidic device that could accurately reproduce the dynamics of vascular anastomosis under treatment of VEGF [53]. All these devices will enable a new generation of studies to investigate the mechanisms of angiogenesis, and provide a novel and practical platform for drug screening.

### 2.3. Hybrid Methods

**Figure 3:**
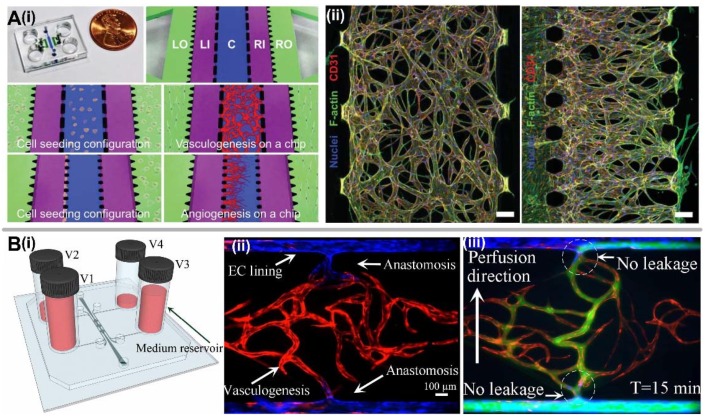
Hybrid methods for in vitro vascularization. (**A**) Vasculogenesis or angiogenesis realized on a single microfluidic device. (**i**) Schematic of microfluidic chip design and cell seeding configurations for vasculogenesis and angiogenesis at different microfluidic channels. (**ii**) Confocal micrographs showing the overall architectures of vascular networks established by vasculogenic and angiogenic processes at day 4. Scale bar, 100 μm. Adapted by permission from Reference [54], copyright Royal Society of Chemistry 2013. (**B**) Advanced vascularization model for generating an intact and perfusable 3D microvascular network. (**i**) Schematic of microfluidic device design by using four medium reservoirs with different hydrostatic pressures. (**ii**) Intact microvascular network formation that incorporates different stages of vascular development including vasculogenesis, EC lining, sprouting angiogenesis, and anastomosis in sequential order. (**iii**) 70 kDa FITC-dextran perfusion confirms physiologic tightness of the EC junctions and completeness of the interconnections between artery/vein and the capillary network. Adapted by permission from Reference [26], copyright Royal Society of Chemistry 2016.

By controlling different factors within the microenvironment, different mechanisms to stimulate vascularization can be achieved on a single microfluidic device. Kim et al. presented a microfluidic device with five parallel microchannels that could generate perfusable microvessel network through either vasculogenesis or angiogenesis by seeding the cells and fibrin gel into different channels (Figure 3A) [54]. For the vasculogenesis process, HUVECs and NHLFs were respectively mixed in fibrinogen solution and introduced separately into the central channel and the stromal cell culture channels. For angiogenesis process, the central channel and stromal cell culture channels were solely filled with fibrin gel, and HUVECs were attached onto the gel wall inside one medium channel contralateral to the NHLF seeding. Recently, Wang et al. developed a versatile microfluidic device design with robust construction methodology to establish an interconnected, perfused vascular network from artery to capillary beds to vein (Figure 3B) [26]. This method combined multiple strategies and vascular development processes such as vasculogenesis, EC lining, angiogenesis, and anastomosis to take place on a single platform in proper sequence. After the formation of a capillary network inside the tissue chamber via vasculogenesis, EC lining along the microfluidic channels adjacent to the tissue chamber was performed to serve as artery and vein. To promote a tight interconnection between the artery/vein and the capillary network, sprouting angiogenesis was induced, leading to anastomosis of the microvascular network inside the tissue chamber and the EC lining along the microfluidic channels. More importantly, this work demonstrated that non-physiologic leakage from the vasculature into interstitial space could be prevented, which was validated by 70 kDa fluorescein isothiocyanate (FITC)-labeled dextran perfusion.

## 3. Vascular Inducing Factors

Microfluidic system has become an emerging tool to develop microvasculature in vitro due to its advantages in precise control of factors within the microenvironment such as fluid flow at the physiological levels, distribution of different chemical factors, and different ECM properties (e.g., stiffness, orientation, etc.). These inducing factors can be divided into three main types: Biomechanical factors, extracellular (or diffusible) signaling molecules, as well as cell source and cell-cell interaction. Table 1 shows the summary of main factors on the influence of different vessel parameters.

### 3.1. Biomechanical Factors

**Figure 4:**
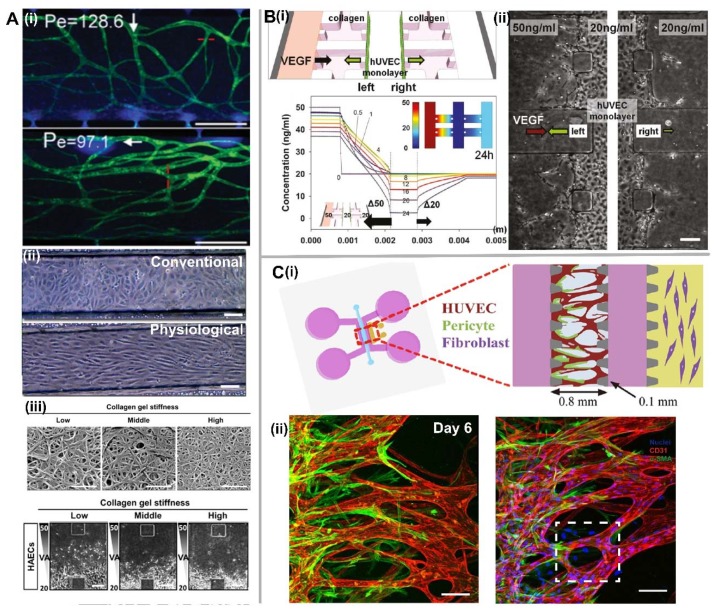
Three major vascular inducing factors in vascularization on chip. (**A**) Biomechanical factors. (**i**) The influence of interstitial flow with high Péclet number on vasculogenesis, and the orientation of microvascular network under the effect of interstitial flow in transverse and longitudinal directions. Scale bar, 500 μm. Adapted by permission from Reference [40], copyright Royal Society of Chemistry 2013. (**ii**) The effect of shear stress on EC lining, and ECs were elongated and aligned with the direction of the flow under the physiological conditions compared with the conventional conditions. Scale bar, 100 μm. Adapted by permission from Reference [57], copyright Nature Publishing Group 2014. (**iii**) The effect of gel stiffness on angiogenesis, and invasion of tubular structures into the stiff collagen gel was significantly reduced compared with the soft gel. Scale bar, 1 μm (top) or 150 μm (bottom). Adapted by permission from Reference [64], copyright Nature Publishing Group 2016. (**B**) Extracellular (or diffusible) signaling molecules. (**i**) Schematic of chip design where HUVECs were cultured in the center channel, and simulation result of VEGF gradient. (**ii**) Apparent sprouting angiogenesis on the left-hand side with a steeper gradient compared with the right-hand side with a gentle gradient. Scale bar, 150 μm. Adapted by permission from Reference [50], copyright American Chemical Society 2011. (**C**) Cell source and cell-cell interactions. (**i**) Schematic of microfluidic device composed of a central vessel channel, two adjacent media channels, and the outermost fibroblast channel. (**ii**) Confocal images showing matured pericytes covered the perfusable EC network on day 6. Scale bar, 100 μm. Adapted by permission from Reference [91], under the Creative Commons Attribution License 2015.

In microfluidic device, biomechanical factors are various forces generated by fluid flow through the microfluidic channel or the ECM. Shear stress is the dominant biomechanical factor that highly depends on the fluid flow. It was found that shear stress would enhance barrier functions by decreasing vascular permeability and narrowing vascular wall to improve stability [12,55,56,57]. For EC lining, ECs were elongated and aligned with the direction of the flow under the effect of shear stress (Figure 4(Aii)). In addition, shear stress has been shown to attenuate HUVEC invasion through nitric oxide (NO) signaling irrespective of interstitial flow direction and the VEGF gradient [58,59]. Galie et al. further concluded that intraluminal shear stress and transmural flow through the endothelium above 10 dyn/cm^2^ can trigger ECs to sprout and invade into the underlying matrix, independent of cell-cell junction maturation or pressure gradient across the monolayer [60].

Interstitial flow is another important regulator of various cell behaviors both in vitro and in vivo [61], and its force imposed on ECs can promote capillary formation. In vivo, interstitial flow in the range of 0.1–1 μm/s can modulate vessel formation [62]. Inside the microfluidic device, the convective velocity in the range of 1.7–11 μm/s with high Péclet number (Pe > 10), defined as the ratio of convective to diffusive transport, can stimulate vasculogenesis regardless of the interstitial flow direction (Figure 4(Ai)) [40]. However, for angiogenesis, new sprouting from an existing vascular network is more active in the reverse direction of interstitial flow [51]. Moreover, it was found that the basal-to-apical transendothelial flow could trigger the transition of ECs from a quiescent to an invasive phenotype to undergo angiogenesis [48].

In addition to fluid forces, hydrogel/ECM mechanical properties such as gel permeability, pore size, fiber diameter and stiffness, and degradation speed also plays an important role in regulating vascular formation [45]. Collagen and fibrin are the most commonly used natural hydrogels, and their mechanical properties can be flexibly fine-tuned. Collagen mechanical properties can be adjusted by changes in temperature or pH while fibrin mechanical properties can be adjusted by changes in fibrinogen and thrombin concentrations. It was found that stiffer gels would generate vessel lumens with small diameters [63], and the invasion of sprouting tubular structures into the stiff collagen gel was significantly reduced compared with the soft gel (Figure 4(Aiii)) [64]. Furthermore, fiber arrangements inside the ECM can also affect vessel alignment in response to contact guidance [65]. In addition to these passive mechanical forces generated by ECM with different mechanical properties, the active mechanical force imposed on ECM will have a profound effect on the microvasculature response. For example, the free-floating scaffolds resulted in randomly orientated vessels. However, the cyclic stretching applied on uniaxially fixated seeded constructs would result in diagonal vessels, whereas the static stretching resulted in vertical vessels [66].

### 3.2. Extracellular (or Diffusible) Signaling Molecules

Growth factors are cell-secreted substances capable of stimulating cellular growth, proliferation, and differentiation. VEGF is the most important growth factor that regulates EC migration and proliferation through VEGF receptor-2 signaling [67]. It was found that VEGF at lower concentrations (2.5–5 ng/ml) induces endothelial sprouting rather than at higher concentrations (15–35 ng/ml) [68]. In addition, negative VEGF gradients result in a process resembling vessel dilation, whereas positive VEGF gradients induce sprouting [59]. Besides VEGF, other angiogenic growth factors such as Angiopoietin 1 (Ang-1), Transforming Growth Factor Beta 1 (TGF-β1), and bFGF can direct angiogenic sprouting from a low-to-high concentration gradient via endothelial tip cell filopodia, and align them into well-organized structures [64,69,70]. Therefore, establishing a concentration gradient of growth factors is crucial for tissue vascularization through either vasculogenesis or angiogenesis in vitro (Figure 4B). This can be achieved by embedding the fibroblast-contained alginate spheroids or the growth factor encapsulated microparticles within the hydrogel to create a local concentration gradient, or by perfusing cell culture media containing growth factors through the microfluidic device [71,72]. Compared to single growth factors acting on the microvascular network, a combination of multiple growth factors can highly enhance vascular density and stability [73,74]. Growth factors with different gradient profiles can also be adjusted by tuning flow rate and designing different microfluidic configurations [75]. For example, a simple device with one central gel channel positioned between two medium channels can generate a linear gradient profile across the gel channel [76]. Besides a linear gradient profile in one direction, two gradient profiles can also be generated orthogonally, which could be used to examine cellular morphogenesis under the influence of multiple well-defined gradients [49]. In addition to the spatial effect of growth factors, temporal change of VEGF concentration could also affect angiogenesis. It was found that an initial gradient of a high VEGF concentration, with a programmed concentration decrease over time, yielded optimal angiogenic sprouting, as compared to a constant dose of VEGF [77]. Therefore, controlled release of growth factors, optimal combination of growth factors, dosage, gradient profile, and appropriate exposure time will facilitate proper vascular formation and stability.

In addition to the direct influence of different growth factors, the oxygen tension may be considered as an indirect method that would affect the secretion of VEGF. It is known that hypoxia inducible factor (HIF)-1 secreted in hypoxic environment can initiate the transcription of VEGF, which plays a critical role in inducing the expression of anti-apoptotic protein, especially in these pathological angiogenesis models such as tumor angiogenesis [78]. In microfluidics, the desired oxygen microenvironment can be generated through different methods. For example, due to the high gas permeability of PDMS used in microfluidics, both hypoxia microenvironment and oxygen tension gradient can be flexibly created by flowing different gases with certain concentration, perfusing gas-equilibrated medium, or loading oxygen scavenging or generation chemicals [79,80]. Furthermore, with the combination of poor gas-permeable thermoplastic, the interference from the external microenvironment can be inhibited, which can improve the oxygen control inside the PDMS-thermoplastic hybrid microfluidic devices [81,82].

### 3.3. Cell Source and Cell-Cell Interaction

ECs are the main cellular component for tissue vascularization. HUVECs have been widely used for microvascular construction in vitro due to their relatively simple isolation process and well-established literature references. However, other EC sources may be used instead of HUVECs for better outcomes or for certain applications that require tissue-specific ECs. For example, it was found that EPCs exhibited a substantial proliferative capacity, which is different from HUVECs with limited proliferation in the absence of pro-angiogenic factors [83,84]. Recently, induced pluripotent stem cells (iPSCs)-derived ECs have become a new EC source for personalized medicine research [85,86,87]. To create a mature and stable microvascular network in vitro, it is necessary to co-culture ECs with mural cells, such as fibroblasts, pericytes or MSCs. Fibroblasts can facilitate EC sprouting and lumen formation by synthesizing and maintaining essential matrix proteins (e.g. Collagen I), as well as secreting various angiogenic growth factors [40,52]. In addition, pericytes and SMCs wrapping around capillary and larger vessels (e.g. arterioles, arteries, venioles, and veins) play an important role in stabilizing newly formed vessels (Figure 4C) [88,89,90,91]. MSCs also exhibit a functional role in vascular stabilization. For example, a co-culture of HUVECs and MSCs derived from either bone marrow or human embryonic stem cells (hESCs) can increase sprouting branches and maintain structural integrity of microvascular network in vitro [76,92].

## 4. Selective Vascular Barrier

**Figure 5:**
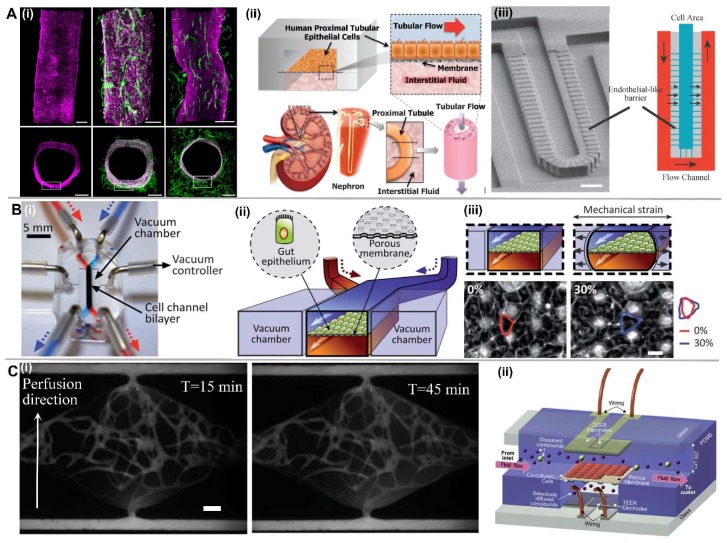
Selective vascular barrier property of different organ chips, construction with porous membrane and permeability characterization methods. (**A**) Microvascular with different structure and selective barrier function in different organ-on-a-chip. (**i**) BBB with tight cell-cell junctions. Fluorescence confocal micrographs of the engineered brain microvessel from human brain microvascular endothelial cells (left), and co-culture with pericytes (middle) and astrocytes (right). Scale bar, 200 μm. Adapted by permission from Reference [95], under the Creative Commons Attribution License 2016. (**ii**) Schematic of a human kidney proximal tubule-on-a-chip in an attempt to mimic the natural architecture, tissue–tissue interface, and dynamically active mechanical microenvironment of the living kidney proximal tubule. Adapted by permission from Reference [96], copyright Royal Society of Chemistry 2013. (**iii**) Schematic of the liver-on-a-chip design that resembles a liver sinusoid, including the endothelial-like barrier layer. Scale bar, 50 μm. Adapted by permission from Reference [99], copyright John Wiley and Sons, Inc. 2007. (**B**) Human gut-on-a-chip. (**i**) Photographic image of the chip prototype. (**ii**) A monolayer of gut epithelial cells is cultured on the flexible porous ECM-coated membrane in the middle of the central microchannel. Vacuum chambers are designed parallel to the channels that can apply mechanical strain to the cell layer. (**iii**) Schematics (top) and phase contrast images (bottom) of intestinal monolayers cultured within the gut-on-a-chip in the absence (left) or presence (right) of mechanical strain (30%; arrow indicated direction) exerted by applying suction to the vacuum chambers. Scale bar, 20 μm. Adapted by permission from Reference [106], copyright Royal Society of Chemistry 2012. (**C**) Vascular permeability characterization. (**i**) Permeability coefficient calculation by measuring perfusion fluorescent solutes across vessel lumen wall over time. Scale bar, 100 μm. Adapted by permission from Ref. [26], copyright Royal Society of Chemistry 2016. (**ii**) Schematic of a multi-layered microfluidic device design with incorporated electrode for internal TEER measurement on a BBB model. Adapted by permission from Reference [93], copyright Royal Society of Chemistry 2012.

ECs exhibit different cellular structure and barrier properties depending on specific organs in the human body. For example, endothelial cells at the blood-brain barrier (BBB) has very tight cell-cell junctions, resulting in very low permeability to protect the central nervous system from toxins and plasma fluctuations (Figure 5(Ai)) [93,94,95]. In kidney, ECs and podocytes form a glomerular filtration barrier that can retain 99.9% of large proteins (Figure 5(Aii)) [96,97]. In contrast, liver sinusoidal ECs have large openings that facilitate bidirectional macromolecular exchange, liposomal transportation, and nitric oxide synthesis (Figure 5(Aiii)) [98,99]. Therefore, to develop organ-on-a-chip systems with tissue-specific characteristics, it is necessary to incorporate a microvascular network with tissue-specific barrier functions. Besides improving the barrier function by imposing shear stress, an increase in circumferential stretch on the vessel wall perpendicular to the direction of flow will enhance endothelial permeability and vascular tone due to the overloaded pressure that is associated with hypertension [100]. In addition to the stimuli with various mechanical factors, the microvascular would react with the loss of barrier function upon the simulation with inflammatory cytokines [11]. Therefore, factors including cell phenotype, perfusion condition, vessel geometry, as well as growing factors and cytokines, will have a significant effect on the vessel permeability.

In versatile organ-on-chips, most designs consist of a vascular barrier made from porous membrane that sandwiched between the blood vessel compartment containing ECs and the other tissue/organ compartment containing other cell types [101,102]. This porous membrane could facilitate the creation of cellular/tissue interface as well as the crosstalk between these two compartments. The porous membrane can be fabricated from a variety of materials with different methods, like the PDMS through the template-based soft lithography [103], polymeric track etched membranes [104], etc. The pore size of porous membrane is likely the most important parameter to affect the cell transmigration, physical contact with the other cells and the paracrine signaling. In addition, the mechanical properties of the porous membrane like stiffness and strain would affect the organization and function of organs as successfully demonstrated in lung-on-a-chip [105] and gut-on-a-chip (Figure 5B) [106]. Furthermore, the surface properties of the porous membrane, functioned as an equivalent to ECM and more specifically the cell basement membrane, would directly stimulate the cell response. It was found that the porous membrane with higher roughness would increase cell adhesion due to more adsorbed protein, while a porous membrane with moderate hydrophilicity would lead to the highest cell adhesion, spreading, and growth. Therefore in order to better mimic the organ-specific vascular barrier properties, it is necessary to integrate a porous membrane with suitable microstructures (pore size, porosity, etc.), mechanical properties (substrate stiffness, strain, etc.), and surface properties (surface roughness and topography, surface chemistry, etc.).

As is known, the tighter endothelial junctions of established tissue barrier correspond to the lower solute permeability. Immunostaining of junctional markers such as platelet endothelial cell adhesion molecule-1 (PECAM-1) and vascular endothelial (VE)-cadherin, as well as the deposition of basement membrane proteins such as laminin and collagen IV are commonly used to validate vascular integrity qualitatively [66]. For quantitative analysis, fluorescent-tagged dextran or other biologically relevant molecules are typically utilized to characterize barrier function by measuring their permeability coefficient across the vessel wall (Figure 5(Ci)) [26,107,108]. Since albumin (MW = 66.5 kDa) is the most common protein in blood plasma, fluorescent-tagged bovine serum albumin (BSA) or 70 kDa dextran are commonly used [108]. Vascular permeability coefficient of microvessels in vitro is typically in the 10^−6^ cm s^−1^ range, which is comparable to the value of tumor microvasculature in vivo [11,17]. In addition to permeability measurement, transendothelial electrical resistance (TEER) is a widely accepted method to quantify vascular integrity by characterizing electric impedance across the endothelium (Figure 5(Cii)) [93,109]. Compared to the molecular permeability test, TEER can be performed in real-time with ease of implementation. However, TEER measurement in self-assembled microvascular models remains a challenge compared to EC monolayer or single-vessel models.

## 5. Application of Engineered Microvascular Networks to Cancer Biology

Besides a better understanding of the mechanisms of vascularization, the other promising application of engineered microvessels is in modeling human diseases in vitro. Endothelial dysfunction is a major physiological mechanism that can cause various vascular diseases such as thrombosis, atherosclerosis, and inflammation [110]. Especially within the field of cancer biology, in vitro tumor models have provided important tools for cancer research and served as low-cost anti-cancer drug screening platforms. Based on the tumor formation type and vascular integration, these models can be broadly classified into four categories: Transwell-based [111], spheroid-based [112], hydrogel droplet embedded culture [113], and vascularized tumor models [114]. More than 90% of cancer-related mortality is attributed to cancer metastasis [115], which often involves multiple steps closely associated with vascular pathology such as tumor angiogenesis [116], intravasation [117], and extravasation [118]. Therefore, engineered vascularized tumor models play an important role in studying cancer metastasis.

### 5.1. Tumor Angiogenesis

**Figure 6:**
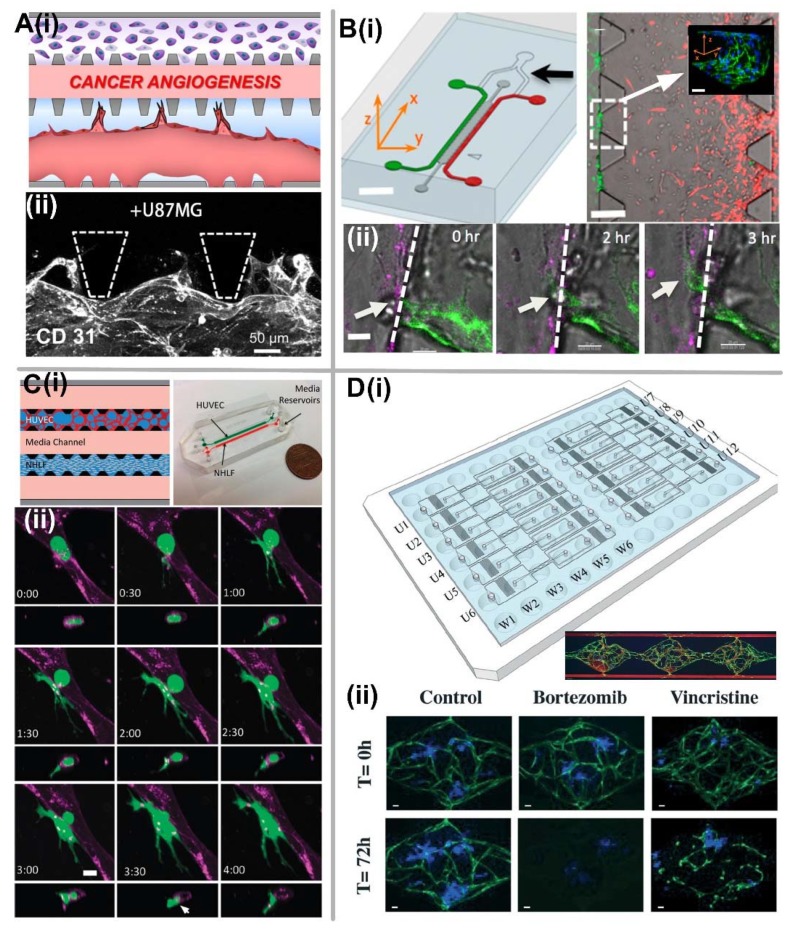
Vascularized tumor-on-a-chip for studying tumor metastasis and anti-cancer drug screening. (**A**) Tumor angiogenesis. (**i**) Schematic of chip design for tumor angiogenesis. (**ii**) 3D confocal image (CD31) shows the angiogenic sprouts stemmed from the microvessel wall grow toward the upper channel stimulated by the pro-angiogenic factors from U87MG cancer cell lines. Adapted by permission from Reference [120], copyright American Institute of Physics 2014. (**B**) Tumor intravasation. (**i**) Schematic of microfluidic tumor-vascular interface model, and the immunostaining image showing confluency of the endothelial monolayer on the 3D ECM. Scale bar, 2 mm (left) or 300 μm (right). (**ii**) Time sequence of a single confocal slice showing a breast carcinoma cell (white arrow) in the process of intravasaton across a HUVEC monolayer. Scale bar, 30 μm. Adapted by permission from Reference [124], copyright National Academy of Sciences, USA 2012. (**C**) Tumor extravasation. (**i**) Schematic and prototype of microfluidic device and cell-seeding configuration. (**ii**) Time-lapse confocal images showing the extravasation process of entrapped MDA-MB-231 (green) from vessel lumen (purple). Scale bar, 10 μm. Adapted by permission from Ref. [126], copyright Royal Society of Chemistry 2013. (**D**) Large-scale anti-cancer drug screening. (**i**) Schematic of microfluidic platform design that custom-fitted into a standard 96-well plate format, and the formation of vascularized microtumor tissue inside three tissue chambers. (**ii**) Drug screening validation with bortezomib and vincristine characterized by the effect on the morphology microvascular network as well as the tumor size. Scale bar, 50 μm. Adapted by permission from Reference [136], copyright Royal Society of Chemistry 2017.

Since the tumor growth can be suppressed by cutting out their nutrient and oxygen supply through tumor vessels, it is essential to have a complete understanding of tumor angiogenesis to develop new cancer therapies. It is known that the rapidly growing angiogenic vasculature around the tumor consequently leads to irregular sprouting, tortuous microstructure, dysfunctional or absent perivascular cells, and leaky barrier properties, all of which are thought to promote tumor metastasis [119]. Several in vitro studies have been performed to study tumor angiogenesis by co-culturing cancer cells and ECs in microfluidic devices. It is normally performed by establishing a vertical 2D endothelial monolayers on the side walls, which can facilitate the better imaging of angiogenic sprouting into the 3D ECM on the axial plane. By attaching ECs to the side wall of fibrin gel, 3D sprouting was promoted by the factors secreted by the highly malignant human glioblastoma, which exhibited aberrant morphology compared to the NHLF-induced sprouts [54]. Similarly, Chung et al. engineered a platform to evaluate and quantify capillary growth and endothelial cell migration from an intact EC monolayer attached onto a collagen gel wall by co-culturing with MTLn3 cancer cells [63]. They observed that cancer cells could either attract ECs and induce capillary formation or have minimal effect, while SMCs suppress endothelial activity. Furthermore, Lee et al. presented a tumor angiogenesis model to quantify angiogenic sprouting from vasculogenesis-derived microvessels (Figure 6A) [120]. It was found that low flow velocities commonly exist in tumor vasculature, suggesting high shear stress regulation of angiogenic activity was absent in these vessels, thereby driving tumor angiogenesis due to the increased tumor-expressed angiogenic factors and endothelial permeability [121]. To further study the complex multicellular interactions during the tumor angiogenesis, a pre-vascularized tumor spheroid model was developed by co-culturing of tumor cells, ECs, and fibroblasts embedded in fibrin gel, which exhibited the robust sprouting angiogenesis after seven days of culture [122]. In addition, different from physiological angiogenesis, tumor angiogenesis is mainly stimulated by the release of HIF-1 due to the excessive cell proliferation and dysfunctional vasculature to initiate transcription of VEGF to stimulate vascular outgrowth toward the tumor tissue [78]. DelNero et al. developed the oxygen-controlled 3D alginate-based tumor model to study the tumor angiogenesis in hypoxic conditions, which demonstrated that the pro-inflammatory pathways were a critical regulator for tumor angiogenesis under the hypoxia microenvironment [123].

### 5.2. Tumor Intravasation

Tumor intravasation is the invasion of cancer cells through the basal membrane into blood or lymphatic vessels near the tumor stroma that can be trigged by chemotactic gradient, oxygen tension, and impaired endothelial barrier function [117]. Zervantonakis et al. demonstrated a model to explore the relationship between cancer cell intravasation and endothelial permeability in the context of cytokine-induced endothelial cell activation and paracrine signaling loops involving macrophages and tumor cells (Figure 6B). They found that macrophage-secreted tumor necrosis factor alpha (TNF-α) could enhance breast cancer cell intravasation due to impaired endothelial barrier function [124]. Similarly, Wong et al. presented a platform that positioned metastatic cancer cells next to artificial, EC-lined microvessels embedded in the ECM. Using real-time live cell fluorescence microscopy, they observed a variety of tumor-endothelium interactions, including invasion, intravasation, angiogenesis, and tumor cell dormancy [125].

### 5.3. Tumor Extravasation

Tumor extravasation refers to circulating tumor cells (CTCs) transmigrating through the endothelium and lodge at the secondary organs. Chen et al. developed a microfluidic platform to study tumor cell extravasation from vasculogenesis-derived microvascular networks (Figure 6C) [126]. Using high resolution time-lapse microscopy, they observed a highly dynamic nature of extravasation events, beginning with thin cancer cell protrusions across the endothelium and followed by extrusion of the remaining cell body through small openings in the endothelial barrier, which grow in size to allow nuclear transmigration. They also found that tumor transendothelial migration efficiency was significantly higher in trapped cells compared to non-trapped cells, and in cell clusters versus single cancer cells. Furthermore, Jeon et al. developed a microfluidic 3D in vitro model to analyze organ-specific human breast cancer cell extravasation into bone and muscle-mimicking microenvironments through a microvascular network concentrically wrapped by mural cells [127]. They found that the extravasation rates of breast cancer cells were significantly higher in the bone-mimicking microenvironment compared to the control matrices without stromal cells, or the muscle-mimicking microenvironments. This result validated a widely accepted phenomenon that specific tumor types preferentially metastasize to specific organ systems.

### 5.4. Tumor Microenvironment

Tumor microenvironment (TME) plays an important role during the tumor metastasis process, which can be broadly classified into biophysical cues (e.g., intramural flow, interstitial flow, and ECM) and biochemical cues (e.g., molecular gradient of growth factors, cytokines, oxygen, and metabolites). For intramural flow in the bioengineered microvessel models, a critical method is to develop a perfusable and functional microvascular network with either EC lining or vasculogenesis/angiogenesis-based methods. The tumor intravasation/extravasation processes are mainly regulated by the alteration of vessel wall permeability and integrity under the effect of shear stress induced by the intramural flow inside vessel lumen. Therefore, the tumor invasion and metastasis events are unlikely to occur in large vessels like arteries and veins due to the high shear stress along the tight and thick vessel wall. In contrast, the capillaries with a smaller size would easily trap the tumor cells, which are more likely to penetrate through the capillaries due to the thinner walls and lower shear stresses [128]. The interstitial flow in TME can lead to the spatial gradient of secreted cytokines, which would contribute to the directional tumor cell migration along/against the interstitial flow direction [129]. Besides the biological flow, the properties of ECM in TME are the other major biophysical factors to affect the tumor cell behavior, including stiffness, pore size, and fiber alignment [130]. It was found that the ECM with both stiff property and large pore size would facilitate the tumor cell migration to promote its invasion events. Furthermore, the hollow microchannel inside ECM remodeled by the MMP can serve as a highway to speed up the tumor cell migration [131]. Similarly, the well aligned hydrogel fibers can also be regarded as the hollow micro-sized tunnels, which can be realized by using the shear stress on the un-polymerized hydrogel inside a narrow microfluidic microstructure or directly applying the mechanical stretching force on the polymerized hydrogel.

Chemokines play a paramount role in the tumor progression. Both tumor cells and stromal cells elaborate chemokines and cytokines. These act either by autocrine or paracrine mechanisms to sustain tumor cell growth, induce angiogenesis and immune response to the tumor [132]. By using microfluidic systems, well-controlled biochemical gradient profiles can be established. Furthermore, due to the diminished supply of nutrients and oxygen caused by the defective vascular with heterogeneous perfusion in fast-growing solid tumor tissue, the TME is often hypoxic. Therefore, the oxygen tension is another critical chemical factor in TME.

### 5.5. Application of Vascularized Tumor-on-a-Chip

The main application for in vitro vascularized tumor models is anti-cancer drug screening. Current drug screening methods still heavily rely on 2D cell culture assays or animal models. While 2D cell culture assays fail to replicate the 3D structure and complexity of tissues in vivo, animal models fail to capture the human-specific response to drugs due to intrinsic differences in anatomy and physiology [133]. While 3D tumor spheroids can address some of these shortcomings, many tumor cell types, especially those with a highly invasive phenotype, cannot readily form spheroids. Since in vivo most drugs are delivered to the targeted tissue through the circulatory system rather than directly imposed on the cultured tissue, incorporating the vasculature into tumor models can better mimic drug delivery to provide the precise information like drug dosage as reference in further clinical trials. Besides the vascularized tumor tissue, co-culture with other stromal cells is another important aspect to mimic the drug delivery microenvironment. It was found that the co-culture of stromal cells and tumor cells showed a significantly higher drug resistance, as compared to the culture only with tumor cells [134]. Sobrino et al. developed a 3D microtumor model supported by perfused human microvasculature for anti-cancer drug screening [135]. They found that the microvessels in this platform are sensitive to the effects of both anti-angiogenic (Pazopanib, Linifanib, Cabozantinib, Axitinib, etc.) and vascular disrupting agents (Vincristine, Taxol). Therefore, this vascularized tumor model can be used to identify drugs that target cancer cells directly, or indirectly through effects on the vasculature to starve off tumor growth. Based on this work, Phan et al. further developed an arrayed vascularized microtumor platform for large-scale drug screening applications and successfully demonstrated its capacity to identify both anti-angiogenic and anti-cancer drugs from a small library of compounds (Figure 6D) [136].

The widely used strategies for anti-angiogenic therapies are to destroy the tumor vasculature, thereby depriving the tumor of nutrients. However, these methods will also severely compromise the delivery of oxygen to the solid tumor, which induces hypoxia microenvironment to make many chemotherapeutics and radiation less effective. In addition, the interstitial hypertension and nonuniform blood flow induced by the abnormalities and leakiness of tumor vasculature will also accumulate the drugs and oxygen at the concentrated region far from the solid tumors, no matter how much therapeutics and oxygenation are pumped into it. Therefore, another concept for tumor anti-angiogenic therapy is to study the normalization of tumor vasculature, which can temporarily “normalize” the abnormal structure and function of tumor vasculature into the functionally normal blood vessels by using certain antiangiogenic agents to make it more efficient for oxygen and drug delivery [137]. The microfluidics-based vascularized tumor model can provide a better understanding of tumor vessel normalization at the molecular, cellular, and functional organ level. Besides the anti-angiogenic therapies, immunotherapies against cancer have shown enormous potential, leading to the recent FDA approval of several drugs that reduce cancer progression [138]. Therefore, more studies on cancer-immune cell interactions should be better characterized by using the vascularized tumor model perfused with immune cells [139].

## 6. Conclusions and Future Perspectives

In this review, we have provided an overview of different vascularization strategies based on microfluidic technology. These strategies can be classified into three categories: EC lining-based methods, vasculogenesis and angiogenesis-based methods, and hybrid methods. It is apparent that each strategy carries both advantages and limitations. While the EC lining-based methods enable immediate media perfusion with well-controlled biomechanical factors, it is inefficient and only suitable to create larger blood vessels based on the predefined microchannel dimensions. In contrast, while vasculogenesis and angiogenesis-based methods provide very limited microenvironment control, they are suitable to create a capillary network down to a few micrometers in diameter by utilizing natural morphogenic properties of ECs. Therefore, an ideal strategy for proper tissue vascularization may lie in the combination of more than one technique to realize the hybrid methods. Moreover, multiple vascular inducing factors, such as biomechanical factors, extracellular (or diffusible) signaling molecules, cell source, and cell-cell interaction should be integrated into more complex microfluidic designs simultaneously to closely mimic the native microenvironment in different organs with specific vascular barrier functions. Furthermore, engineered microvessels also play an important role in modeling various human diseases, especially for the cancer research. The construction of vascularized tumor-on-a-chip can serve as a powerful tool to study the mechanisms of cancer metastasis at different stages like tumor angiogenesis, intravasation, and extravasation as well as their potential applications in anti-cancer drug screening and normalization of tumor vessels.

While many proof-of-principles studies have demonstrated versatile vascularization strategies and its applications in tumor models, challenges still remain to be solved. For example, there is an urgent need to establish more precise and geometrically controlled methods with more dynamic and flexible on-chip microenvironment control to recapitulate all aspects of vasculature in vivo. To construct a functional vascularized microtissue, interactions between ECs, supporting mural cells, and organ-specific cells with in vivo-relevant cell densities are required. Additionally, current culture media are optimized for a specific cell type when cultured in monolayer. Once these cells are in a complex microenvironment, it is necessary to have a universal blood substitute to equally supplement multiple cell types. Another major consideration is to develop thick, implantable vascularized tissue constructs from microns to millimeters with synthetic biocompatible and degradable scaffold materials for tissue repair or regeneration, as described in the recent AngioChip design [140]. The exciting future application for engineered microvessels is their potential use in the field of precision medicine by creating patient-specific microvessel models using ECs derived from iPSCs. The future work for vascularized tumor models may lie in the better understanding of the tumor microenvironment, and how to manipulate different microenvironment factors to affect the cancer progression. Besides the vascular inducing factors, the solid tumor stress induced by the abnormally high amounts of collagen and other proteins is also the key factor to design the physiologically relevant in vitro vascularized tumor models. For drug screening applications, it is imperative to develop automated, high throughput, reproducible, cost-effective, and easy-to-use microfluidic 3D cell culture systems. In terms of device design, it is necessary to integrate multiple on-chip detection sensors and analysis components for real-time monitoring and analysis of growing tissues. Finally, while the majority of organ-on-a-chip platforms are fabricated using PDMS and its derivatives due to their low cost, easy-to-use, and biocompatibility, they can absorb small molecules with transient mechanical properties, making them ineffective for long-term cell/tissue culture and drug studies. Therefore, there has been a push towards thermoplastic materials (like polystyrene, cyclic olefin copolymer, and polycarbonate, etc.) to replace PDMS for organ-on-a-chip platforms [141].

To summarize, although significant progress has been made towards tissue vascularization and related vascularized tumor models in the past decade, numerous challenges remain and will be addressed in future studies with a combination of microfluidics, microfabrication, biomaterials, tissue engineering, and other related technologies. Thus, a multidisciplinary, collaborative approach between engineers and biologists is required to develop more novel microphysiological systems that can closely mimic organ-specific physiology in vivo.

## Figures and Tables

**Table 1:** Main factors on the influence of different vessel parameters.

Factors	Vessel Parameters	Effect
Shear stress	Barrier function	Decreasing permeability [12,55,56,57]
	Cell orientation	Elongated and aligned along flow direction in EC lining [57]
	EC invasion	Attenuate invasion [58,59]
Interstitial flow	Vasculogenesis	Promote capillary formation regardless of flow direction [40]
	Angiogenesis	More active in reverse direction of interstitial flow [51]
Stiff ECM	Vessel lumen	Small diameter [63]
	Sprouting invasion	Significantly reduced [70]
Fiber arrangements of ECM	Vessel alignment	Along the fiber orientation [64]
VEGF concentration	Endothelial sprouting	Induce sprouting at low concentration (2.5–5 ng/mL) [67]
VEGF gradient	Vessel morphology	Positive gradient induces sprouting, and negative gradient induces vessel dilation [59]
Hypoxia	Angiogenesis	Promote angiogenesis, such as tumor angiogenesis [78]
Co-culture with fibroblast	Perfusable vascular formation	Promote EC sprouting and lumen formation [40,52]
Co-culture with mural cells	Vessel stabilization	More stable [88,89,90,91]

## Abbreviations

The following abbreviations are used in this manuscript:

3D	Three-dimensional
2D	Two-dimensional
ECs	Endothelial cells
PDMS	Polydimethylsiloxane
HUVECs	Human umbilical vein endothelial cells
PEG	Polyethylene glycol
SMCs	Smooth muscle cells
ECM	Extracellular matrix
EPCs	Endothelial progenitor cells
ECFC-ECs	Endothelial colony forming cell-derived endothelial cells
NHLF	Normal human lung fibroblast
VEGF	Vascular endothelial growth factor
bFGF	Basic fibroblast growth factor
MMPs	Metalloproteinases
DAPI	4′,6-diamidino-2-phenylindole
FITC	Fluorescein isothiocyanate
NO	Nitric oxide
Ang-1	Angiopoietin 1
TGF-β1	Transforming growth factor beta 1
HIF	Hypoxia inducible factor
iPSCs	Induced pluripotent stem cells
MSCs	Mesenchymal stem cells
hESCs	Human embryonic stem cells
BBB	Blood-brain barrier
PECAM-1	Platelet endothelial cell adhesion molecule-1
VE	Vascular endothelial
BSA	Bovine serum albumin
TEER	Transendothelial electrical resistance
TNF-α	Tumor necrosis factor alpha
CTCs	Circulating tumor cells
TME	Tumor microenvironment
